# The Role of ST2 Receptor in the Regulation of *Brucella abortus* Oral Infection

**DOI:** 10.3390/pathogens9050328

**Published:** 2020-04-28

**Authors:** Raiany Santos, Priscila C. Campos, Marcella Rungue, Victor Rocha, David Santos, Viviani Mendes, Fabio V. Marinho, Flaviano Martins, Mayra F. Ricci, Diego C. dos Reis, Geovanni D. Cassali, José Carlos Alves-Filho, Angelica T. Vieira, Sergio C. Oliveira

**Affiliations:** 1 Department of Genetics, Institute of Biological Sciences, Federal University of Minas Gerais—Belo Horizonte, Minas Gerais 31270-901, Brazil; raianyas@yahoo.com.br; 2 Department of Biochemistry and Immunology, Institute of Biological Sciences, Federal University of Minas Gerais—Belo Horizonte, Minas Gerais 31270-901, Brazil; pccampos78@gmail.com (P.C.C.); marcellarungue@hotmail.com (M.R.); victormelobio@gmail.com (V.R.); davidmartinsst@gmail.com (D.S.); mendesviviani.a@gmail.com (V.M.); fabiovitarelli@yahoo.com.br (F.V.M.); angelicathomaz@gmail.com (A.T.V.); 3 Department of Microbiology, Institute of Biological Sciences, Federal University of Minas Gerais—Belo Horizonte, Minas Gerais 31270-901, Brazil; flaviano@icb.ufmg.br; 4 Department of General Pathology, Institute of Biological Sciences, Federal University of Minas Gerais—Belo Horizonte, Minas Gerais 31270-901, Brazil; riccimayra@gmail.com (M.F.R.); diegobiomed.reis@gmail.com (D.C.d.R.); geovanni.cassali@gmail.com (G.D.C.); 5 Department of Pharmacology, Ribeirao Preto Medical School, University of Sao Paulo, Ribeirao Preto 14049-900, Brazil; jcafilho@usp.br

**Keywords:** ST2 receptor, *Brucella abortus*, oral infection

## Abstract

The ST2 receptor plays an important role in the gut such as permeability regulation, epithelium regeneration, and promoting intestinal immune modulation. Here, we studied the role of ST2 receptor in a murine model of oral infection with *Brucella abortus*, its influence on gut homeostasis and control of bacterial replication. Balb/c (wild-type, WT) and ST2 deficient mice (ST2^−/−^) were infected by oral gavage and the results were obtained at 3 and 14 days post infection (dpi). Our results suggest that ST2^−/−^ are more resistant to *B. abortus* infection, as a lower bacterial colony-forming unit (CFU) was detected in the livers and spleens of knockout mice, when compared to WT. Additionally, we observed an increase in intestinal permeability in WT-infected mice, compared to ST2^−/−^ animals. Breakage of the intestinal epithelial barrier and bacterial dissemination might be associated with the presence of the ST2 receptor; since, in the knockout mice no change in intestinal permeability was observed after infection. Together with enhanced resistance to infection, ST2^−/−^ produced greater levels of IFN-γ and TNF-α in the small intestine, compared to WT mice. Nevertheless, in the systemic model of infection ST2 plays no role in controlling *Brucella* replication in vivo. Our results suggest that the ST2 receptor is involved in the invasion process of *B. abortus* by the mucosa in the oral infection model.

## 1. Introduction

Brucellosis is a worldwide zoonotic disease caused by facultative intracellular pathogen of the genus *Brucella* [1]. Bacteria of the genus *Brucella* infect a wide variety of land and aquatic mammals, including pigs, cattle, goats, sheep, dogs, dolphins, whales, seals, and desert wooden mice. Traditionally, the genus *Brucella* consisted of six recognized species, grouped according to their primary host preferences, i.e., *B. abortus*, bovine; *B. melitensis*, sheep and goats; *B. suis*, pigs; *B. ovis*, sheep; *B. kennels*, dogs; and *B. neotomae*, desert wood mice. Recent new species were isolated from humans (*B. inopinata*), aquatic mammals (*B. pinnipedialis* and *B. ceti*), and from a common rat (*B. microti*), raising the current number to 10 species of the genus [2]. Human brucellosis can be mainly caused by *Brucellaabortus* and *Brucella melitensis*, leading not only to cases of morbidity but also severe economic losses caused mainly by abortions and infertility in infected animals [3]. The natural infection by *Brucella* occurs mainly by the oral and nasal routes through consumption of raw milk and unpasteurized dairy products from infected animals, inhalation of aerosols containing the pathogen, contact with infected animals and their secretions, and by the habit of cattle to lick and smell newborn animals or even aborted fetuses. In addition, there is laboratory and occupational contamination, affecting researchers, farmers, slaughterhouse workers, butchers, and veterinary doctors (many cases of accidental self-inoculation of the vaccine against animal brucellosis), and there are forms (although very unlikely) of human transmission such as contamination of plants by feces and urine from infected animals, and breastfeeding [4,5,6,7].

Brucellosis is a systemic disease in which any organ or tissue of the body might be involved. Affected individuals present nonspecific symptoms shared with several other diseases, which cause human brucellosis to present underestimated data of epidemiological distribution [8]. In humans, the main symptoms of the acute phase of the disease are undulating fever, headaches, fatigue, myalgia, and weight loss. In the chronic phase of the disease endocarditis, arthritis, osteomyelitis, and neurological complications can be observed [9]. In animals, brucellosis is a chronic infection that persists throughout life. In females, *Brucella* causes tropism through the bovine placental hormone, erythritol, leading to lesions in the uterine glands, while in males, the bacterium causes tropism through male hormones like testosterone, addressing the testicles. Thus, *Brucella* infection primarily affects the reproductive organs causing abortion and infertility [10].

*Brucella* spp. can resist death by neutrophils, and replicate within macrophages and dendritic cells, thus, maintaining a long lasting interaction with the host cells [11]. Therefore, innate immunity has developed important mechanisms for detecting and eliminating these bacteria. Toll-like receptors (TLRs) have already proven to be important in the control of *Brucella abortus* infection. The recognition of *B. abortus* molecules by TLR2 (external membrane proteins, Omp16 and Omp19), TLR4 (*Brucella* LPS and *Brucella* lumazine synthase), and TLR9 (*Brucella* DNA), activates intracellular signaling via MyD88, resulting in the activation of NF-κB, MAP kinases, and expression of pro-inflammatory cytokines [12,13,14,15,16]. TLR2 does not participate in the in vivo control of infection, contributing only to the production of pro-inflammatory cytokines [12,14]. However, TLR9 has played a prominent role in relation to in vivo and in vitro control of *B. abortus* infection [17]. In addition to the receptors mentioned above, *B. abortus* leads to activation of NLRP3 (through reactive mitochondrial oxygen species induced by bacteria) and AIM2 (recognition of bacterial DNA) inflammasomes, leading to activation of innate immunity and infection control [18]. The STING protein was also determined as an important adapter molecule required for resistance against this bacterium [19,20].

In the context of intestinal immunity, it is important to highlight the importance of these receptors in this microenvironment, since several of these innate immunity receptors such as the TLRs, NRLs, G protein-coupled receptors (GPCRs), and STING are also expressed in the intestinal mucosa, having an important function in the maintenance of host commensal microbiota and intestinal homeostasis [21,22,23]. Among these innate immunity receptors, the ST2 receptor and its ligand, cytokine IL-33 has been widely studied since its discovery [24]. The ST2 receptor of the IL-1 family, also called IL1rl1, tumorigenicity suppressor 2, growth stimulation expressed in gene 2 and serum stimulation 2, was classified as a receptor for IL-33 in 2005 [25,26].There are four isoforms encoded by the ST2 gene. The two most prominent isoforms include the ST2L transmembrane, which acts as a membrane receptor, responsible for binding the IL-33 and activating the signaling cascades to improve the functions of the cells that express this receptor, and the sST2, presented in a soluble form, which acts by sequestering the free IL-33, preventing its signaling. They are the consequence of a double system of promoters (sST2 proximal promoter and ST2L distal promoter) that results in the differential expression of mRNA. ST2L, like other IL-1 receptors, consists of an extracellular domain, transmembrane domain and cytoplasmic domain (Toll/interleukin-1 receptor (TIR)), while sST2 does not have the transmembrane and cytoplasmic domains and therefore exists as a soluble protein. In addition, alternative splicing results in the formation of ST2V and ST2LV. ST2V shares the same extracellular and transmembrane domain as ST2L, but is remarkable for its unique hydrophobic tail and is particularly enriched in the gastrointestinal tract. Finally, ST2LV notably does not have the ST2L transmembrane domain, but maintains the intracellular domain [27,28].The ST2 receptor is expressed in a wide variety of immune cells, such as conventional T cells, particularly regulatory T cells (T regs) [29], innate type 2 lymphoid cells (ILC2) [30], polarized macrophages M2 [31], eosinophils [32], basophils [33], neutrophils [33], NK cells [34], iNKT cells [34], and several other immune cells and their soluble isoform.sST2 can be produced spontaneously by the small intestine.

As an alarmine, IL-33 is one of the first molecules that “sounds the alarm” to indicate that there has been a violation of the primary defenses of the intestinal epithelium against pathogens and other threats [35]. IL-33 is produced by a variety of stromal cells and organ parenchyma, such as smooth muscle cells, fibroblasts, myofibroblasts, endothelial cells, glia cells, osteoblasts, adipocytes, and by different cells of the immune system, such as macrophages, dendritic cells, and mast cells [36]. IL-33 acts on several cell types, including cells of hematopoietic origin and non-hematopoietic cells. The secretion of IL-33 has been described in monocyte lineage (THP-1 cells), in response to different stimuli—bacterial infection, lipopolysaccharide (LPS) with aluminum adjuvant, and isolated LPS. [29,32]. In order to maintain the integrity of this mucosal barrier, the intestinal epithelium undergoes rapid and continuous self-renewal to replace the damaged cells. Activation of the IL-33/ST2 pathway in epithelial progenitor cells leads to inhibition of Notch signaling and results in differentiation of stem cells towards a line of secretory intestinal cells [37], resulting in the production of mucin, an important barrier mechanism of intestinal immunity, decreasing the interaction of the intestinal epithelium and pathogenic bacteria [38]. Moreover, the activation of this axis is important to recruit and activate innate immune cells, inducing Th1 or Th2 responses, according to the required immune response [39]. Although brucellosis is a worldwide zoonosis, the mechanisms involved during the course and establishment of the natural oral infection by *Brucella abortus* are still poorly studied. With regard to the process of invasion of *Brucella* through mucosal barriers, there are few studies on the mechanisms involved in the ability of this pathogen to interact with the epithelial cells of the gastrointestinal (GI) tract with the host microbiota, and also with the subsequent immune and homeostatic response in the gastrointestinal tract. The intraperitoneal infection pathway is the most commonly used in studies using the murine model. This route favors the immediate systemic dissemination of *Brucella* and its proliferation in lymphoid tissues, especially in the spleen. However, considering that the oral route is the main route of natural infection in humans and animals, there is a need to understand the mechanisms of the establishment of oral infection, so new therapeutic strategies can be developed in order to control this disease. Since, the IL-33/ST2 axis is positioned to interact with the main components of the intestine, which include epithelial cells in response to cell damage and a microbiome composed of commensal bacteria and immune mucosal cells [24], we investigated the role of the ST2 receptor in the immune response against *Brucella abortus* oral infection.

## 2. Results

### 2.1. The Absence of the ST2 Receptor Confers Partial Resistance to Oral Infection 

**Figure 1:**
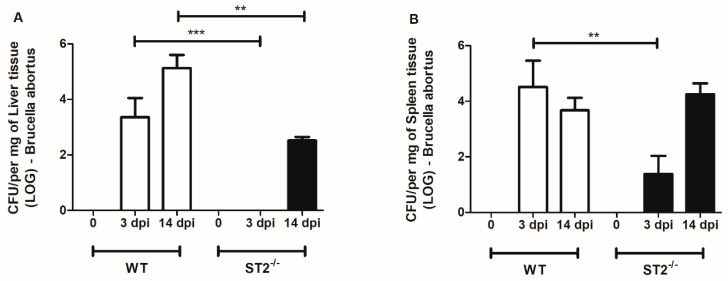
The absence of the ST2 receptor confers partial resistance to oral infection. Wild-type (WT) mice and ST2-deficient mice were orally infected by 1 × 10^9^ colony-forming unit (CFU) of *Brucella abortus* and were sacrificed after 3 and 14 days of infection. The livers (**A**) and spleens (**B**) of the mice were collected and processed for evaluation of the number of viable bacteria through CFU counts. Results expressed as mean ± standard deviation (*n* = 5–7). The data are representative of 3 independent experiments. ** *p* < 0.01, *** *p* < 0.001.

Considering that one of the main routes of the *Brucella* infection is through oral surfaces, we assessed the susceptibility of wild-type (WT) mice and animals deficient for the ST2 receptor (ST2^−/−^) to oral infection with *Brucella abortus*, by determining the number of colony forming units in livers and spleens, 3to 14 days post-infection. We observed higher CFUs of *Brucella* in livers (Figure 1A) and spleens (Figure 1B) of WT mice compared to ST2^−/−^ animals, 3 days after oral infection. Regarding the time of 14 days post-infection, we also observed reduced numbers of bacterial CFUs in livers of ST2^−/−^ mice compared to WT, but not in the spleens of these animals. These findings suggest an enhanced resistance to *Brucella* infection in ST2 knockout mice compared to WT. 

### 2.2. Absence of ST2 Resulted in Change of Intestinal Architecture

**Figure 2:**
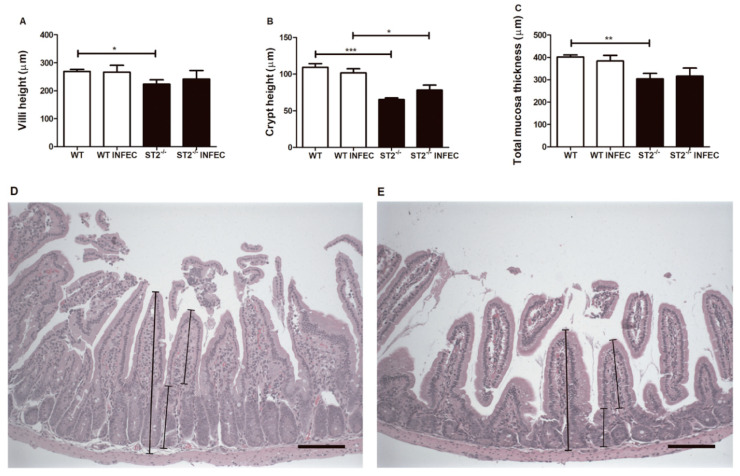
Alterations in mucosa structure in WT and ST2^−/−^ mice during *Brucella* infection. Duodenum of wild-type (WT) and ST2^−/−^ uninfected and infected mice were collected for analysis of (**A**) villi height, (**B**) crypt height and (**C**) total mucosa thickness. Representative photomicrographies of hematoxylin and eosin–stained duodenum sections from WT (**D**) and ST2^−/−^ (**E**) mice evidencing total mucosa thickness, crypt and villi height. Bars represent 100 μm. * *p* < 0.05; ** *p* < 0.01; *** *p* < 0.001.

Intestinal epithelial cells produce antimicrobial effectors that play a central role in shaping the gut microbial community and protecting mucosal tissues from colonization and invasion of commensal microorganisms. To investigate the potential role of ST2 in intestine homeostasis, we analyzed histology sections of small intestine from WT and ST2^−/−^ mice. First, we observed in H&E-stained sections that the villi, crypt, and mucosa thickness of small intestine in naive ST2^−/−^ mice were shorter than in WT animals (Figure 2A–C), regardless of the infection. After oral infection by *Brucella abortus*, we did not observe a major alteration in the gut architecture within each mouse group. Representative photomicrographies of hematoxylin-and-eosin-stained duodenum sections from WT (Figure 2D) and ST2^−/−^ (Figure 2E) are shown. Together, these data indicate that an intact ST2 signaling is important to maintain gut mucosa integrity.

### 2.3. ST2 Receptor is Important in the Maintenance of the Intestinal Epithelial Barrier 

**Figure 3:**
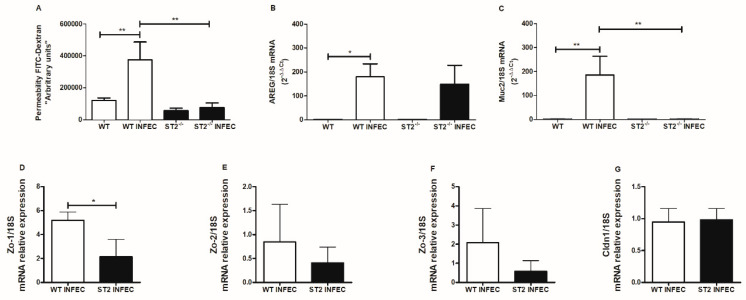
The ST2 receptor is important in the maintenance of the intestinal epithelial barrier and in the transcriptional regulation of Muc2 and ZO-1. WT mice and ST2-deficient mice were orally infected with 1 × 10^9^ CFU of *B. abortus* and after 3 days of infection intestinal permeability was evaluated (**A**). Small bowel samples were also collected for transcriptional analysis of *AREG* (**B**), *Muc2* (**C**), *ZO-1* (**D**), *ZO-2* (**E**), *ZO-3* (**F**), and *claudin-1* (**G**) genes, using qPCR. Results expressed as mean ± standard deviation (*n* = 5–7). The data are representative of two experiments. * *p* < 0.05; ** *p* < 0.01.

The role of the ST2 receptor in maintaining the integrity of the intestinal epithelial barrier following *Brucella infection* was evaluated by the FITC-labeled dextran flow method (Figure 3A). We observed that in the WT animals, *Brucella* infection led to an increased permeability of the epithelial barrier (Figure 3A) (observed by increase of FITC-dextran in the serum of animals). In contrast, ST2^−/−^ mice intestinal permeability was not altered after infection (3 days). We also evaluated the regulation of amphiregulin (AREG) and mucin molecule 2 (MUC2) expression in WT and ST2^−/−^ mice, after 3 days of oral infection with *Brucella*. Amphiregulin and MUC2 are two important components to protect the intestinal epithelium. Regarding the expression of amphiregulin, which is critical for intestinal epithelial regeneration after injury, *B. abortus* infection increased the expression of *AREG* in both WT and ST2^−/−^ mice (Figure 3B). Additionally, we determined that *MUC2* expression following *B. abortus* infection requires ST2 (Figure 3C), suggesting the participation of ST2 in the transcriptional regulation of this molecule. Tight junctions (TJs) play an important role in intestinal function. TJs in intestinal epithelial cells are composed of different junctional molecules, such as claudins, zonula occludens (ZO-1, -2, and -3), among others. Therefore, we determined the role of ST2 in *ZO-1*, *-2*, and *-3* and *claudin-1* expression in intestinal tissue. Herein, we showed that animals lacking ST2 had reduced expression levels of *ZO-1* and to a less extent, that of *ZO-2* and *-3*, when compared to WT mice (Figure 3D,E,F). Regarding *claudin-1* mRNA transcripts, the levels of this TJ remained similar between both mouse groups (Figure 3G).

### 2.4. Lack of ST2 Receptor Modulates the Recruitment of Neutrophils and Eosinophils and Increases the Production of IFN-γ and TNF-α in Small Intestine after Brucella abortus Infection

**Figure 4:**
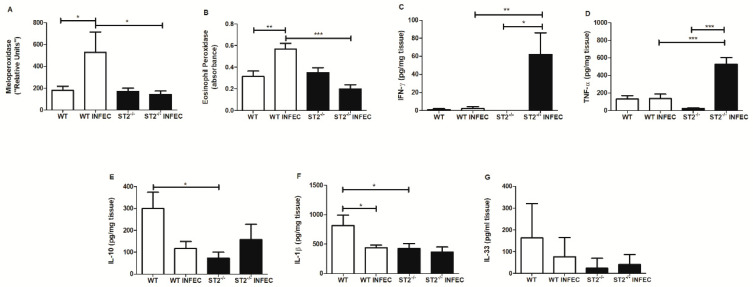
ST2 receptor deficiency modulates the recruitment of neutrophils and eosinophils and increases the production of IFN-γ and TNF-α after *Brucella abortus* infection. WT and ST2^−/−^ mice were infected orally with 1 × 10^9^ CFU of *B. abortus* and after 3 days of infection, small intestine samples were collected for processing and evaluation of myeloperoxidase (**A**) and eosinophil peroxidase (**B**). Tissue samples were also assessed for cytokine production, such as IFN-γ (**C**), TNF-α (**D**), IL-10 (**E**), IL-1β (**F**), and IL-33 (**G**) by ELISA. Results are expressed as mean ± standard deviation (*n* = 5–7). The data are representative of 3 independent experiments. * *p* < 0.05; ** *p* < 0.01; *** *p* < 0.001.

In order to investigate whether the inflammatory response could be involved in the resistance phenotype observed in ST2^−/−^ after *B. abortus* infection, we determined myeloperoxidase (MPO) and eosinophilic peroxidase (EPO) activity as an indirect measurement of neutrophils and eosinophils influx. After *Brucella* infection, there was an increase in MPO (Figure 4A) and EPO (Figure 4B) activity in WT mice, which was not observed in the ST2^−/−^-infected animals, suggesting that the absence of the ST2 receptor somehow modulated the recruitment of neutrophils and eosinophils after infection. Additionally, we also determined the participation of ST2 in the production of cytokines involved in the intestinal immune response, such as IFN-γ, TNF-α, IL-10, IL-1β, and IL-33. The level of these cytokines was measured in small bowel fragments, from non-infected and infected mice after 3 days of infection. Herein, we observed that *Brucella* infection increased the production of IFN-γ (Figure 4C) and TNF-α (Figure 4D) in ST2^−/−^ mice, when compared to the WT animals. This Th1-like profile detected in ST2^−/−^ mice might be related to a reduction in the bacterial load observed in the spleens and livers of these animals, as previously observed by us and others [40,41]. Regarding the production of IL-10 (Figure 4E) and IL-1β (Figure 4F), there is no difference in the levels of these cytokines produced between ST2^−/−^-infected mice when compared to the WT-infected animals. As for IL-33 (Figure 4G), an ST2-binding cytokine, we observed that in ST2^−/−^ mice the production of this cytokine was already naturally decreased and after infection there was no change in this profile when compared to the WT mice.

### 2.5. ST2 Receptor Does Not Play a Role in Systemic Infection Caused by Brucella abortus

**Figure 5:**
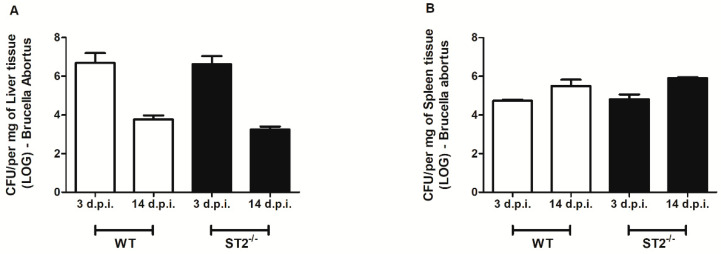
Lack of ST2 receptor does not influence systemic infection induced by *Brucella abortus*. WT mice and ST2-deficient mice were infected intraperitoneally with 1 × 10^6^ CFU of *B. abortus* and sacrificed after 3 and 14 days of infection. The livers (**A**) and spleens (**B**) of these mice were collected and processed for evaluation of the number of viable bacteria through CFU count. Results expressed as mean ± standard deviation (*n* = 5–7). The data are representative of 3 independent experiments.

The resistance or susceptibility phenotype to systemic infection by *Brucella abortus* was evaluated by determining the number of CFU in the livers and spleens of WT versus ST2^−/−^mice, after 3 and 14 days of intraperitoneal (i.p.) infection. We observed that the bacterial load was similar in livers (Figure 5A) and spleens (Figure 5B) of WT and ST2^−/−^ mice after infection. These findings suggest that lack of ST2 plays no role in *Brucella* control in vivo, after intraperitoneal infection.

### 2.6. The Absence of the ST2 Receptor Does Not alter the Production of Nitric Oxide by Macrophages

**Figure 6:**
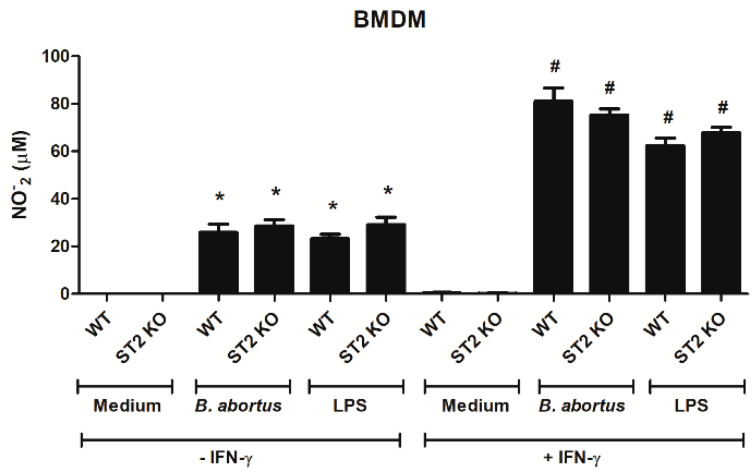
The absence of the ST2 receptor did not alter the production of nitric oxide by macrophages. Macrophage was derived from WT and ST2-deficient mice bone marrow and stimulation with *Brucella abortus* or lipopolysaccharide (LPS) was performed in the presence or absence of IFN-γ. The supernatant was collected to perform the Griess assay, as already described. * *p* < 0.001 when compared to the medium. # *p* < 0.001 when compared to the cells with no IFN-γ. Results expressed as mean ± standard deviation (*n* = 5–7). The data are representative of two independent experiments.

To evaluate the nitric oxide (NO) production in WT and ST2^−/−^ macrophages, and to correlate it with the potential microbicide activity, nitrite, a stable metabolite of NO, was measured using Griess reagent on macrophage supernatants. We observed that the production of NO in the macrophages of both mouse strains when stimulated with *Brucella* or LPS is similar, in the presence or absence of IFN-γ (Figure 6). Therefore, our findings suggest that ST2 deficiency does not influence the ability of *Brucella* infected macrophages to produce NO.

## 3. Discussion

Infections caused by the bacteria of the genus *Brucella* were mainly transmitted orally to human and animals. *Brucella* has a rapid capacity for infectivity in the oral infection model (by gavage or inoculation in the oral cavity), where after one hour of infection, bacteria were already found in the lumen and in the epithelium of the duodenum [42]. Few virulence factors of *Brucella* that are important for the establishment of infection through the oral route have been described, such as urease [43], which confers resistance to gastric acidity, cholylglycine hydrolase (CGH) [44] which induces resistance to bile salts, and the *Brucella* protease inhibitor Omp19 [42] which induces resistance to the action of proteases.

When gastrointestinal tract defense cells fail to capture microorganisms, they are drained mainly through the portal vein into the liver [45]. Previous studies have shown that in bacterial infections, higher concentrations of LPS are detected in the portal vein when compared to other hepatic or peripheral veins and, interestingly, bacteria can be cultivated even from healthy liver explants [46,47]. The phenotype of resistance exhibited by ST2-receptor deficient mice during oral infection was lost when intraperitoneal infection was performed. Considering that the portal vein might be the main route for systemic dissemination of *Brucella*, we first speculated that the liver from ST2-deficient mice might be mounting its own immune response and consequently, decreasing the number of viable bacteria and their ability to spread systemically. However, when we measured IFN-γ and TNF-α production by liver cells, ST2 knockout and WT mice produced similar levels of these cytokines (Figure S1 found in the Supplementary Materials). Therefore, we suggest that other mechanisms might be involved in reduced bacterial counts observed in ST2^−/−^ livers.

The gut-associated lymphoid tissue (GALT), such as the Peyer’s patches (PPs) along with the intestinal mucosal epithelium, act as a sentinel for recognition and initiation of immune responses against pathogenic bacteria [48]. The process of invasion of *Brucella* into the gastrointestinal tract occurs through its ability to translocate via M cells, which occurs by interaction with the prionic protein PrP^c^ that are highly expressed on the apical surface of these cells [49]; however, this process does not lead to the rupture of the cell–cell junctions [50]. Another mechanism related to the invasion process is through the intestinal epithelial cells [51], but this mechanism has not yet been fully clarified. Tight junctions (TJs) play an important role in intestinal function. TJs in intestinal epithelial cells are composed of different junctional molecules, such as claudins, zonula occludens (ZO-1, -2, and -3), and occluding, among others. In this study, we determined the role of ST2 in *ZO-1, -2*, and *-3* and *claudin-1* expression in intestinal tissue. Herein, we showed that animals lacking ST2 had reduced expression levels of *ZO-1* and to a less extent that of *ZO-2* and *ZO-3*, when compared to WT mice. Regarding *claudin-1* mRNA transcripts, the levels of this TJ remained similar between both mouse groups. The reduced expression of zonula occludens (ZO) molecules might not have a direct relationship to intestinal permeability in this model since ST2^−/−^ mice had reduced intestinal permeability, compared to WT animals, as measured by the FITC-dextran method. Rather, diminished expression of these tight junction gene products might correlate with enhanced IFN-γ production observed in ST2^−/−^ animals, as recently demonstrated in the *Salmonella enteritis* infection model [52]. Breaking the epithelial barrier after oral infection resulted in increased intestinal permeability observed in WT mice and could be one important mechanism that facilitates the entry and spread of this pathogen. Studies using a model of ex vivo infection in the ileal bowel loop showed that the migration of *Brucella* through the intestinal epithelium occurs via endocytosis by the follicle-associated epithelium (FAE) in Peyer’s patches or by its uptake by the penetrating dendritic cells of the FAE [49,53]. Additionally, Rosseti and collaborators (2013) [54] observed through microarray analysis that two pathways related to the intestinal epithelial barrier were repressed during the initial phase of *Brucella* infection, suggesting the subversion of the barrier function and facilitating transepithelial migration. Thus, a variety of pathogens use molecules involved in cell adhesion and invasion, such as the *Helicobacter pylori*, whose type IV secretion system injects one of its effectors (CagA) into the host cell, modifying several processes and culminating in the rupture of the epithelial barrier and invasion of the bacteria [55]. Although, we did not explore the infection of *Brucella abortus* in intestinal epithelial cells in vitro, the breaking of the epithelial barrier in vivo might be associated with the presence of the ST2 receptor, since no increase in intestinal permeability was observed in ST2 knockout mice after infection, when compared to WT animals.

Another mechanism related to the process of maintaining the epithelial barrier, involves amphiregulin (AREG), which plays a role in intestinal epithelial regeneration after injury [56] and in cellular proliferation [57]. The level of expression of this molecule after infection was similar in both animals analyzed, suggesting that the change in intestinal permeability observed in WT mice is not mediated by the participation of ST2 in transcriptional regulation of *AREG*. The intestinal mucus is one of the main components of defense against invasion of pathogens and protects the epithelium from physical damage. Muc2 mucin is produced and secreted by intestinal goblet cells. We believe that the increased expression of MUC2 in WT mice might be linked to augmented intestinal permeability. Recently, a higher expression level of the mucin glycoprotein Muc2 in enteroids following *Shigella flexneri* infections was reported [58]. These findings suggest that mucus production might not be an important factor involved in the phenotype of decreased intestinal permeability observed in ST2 knockout-infected mice, and that other mechanisms are involved in the intestinal barrier of ST2^−/−^ animals. 

During infections, depending on the organ involved, IL-33/ST2 signaling might induce the necessary immune response to control the infectious foci, which might be a Th1 or Th2 type of response [38]. The increase of IFN-γ and TNF-α after the infection observed in the knockout mice might be associated with a greater defense of the intestine against the invasion of *B. abortus*, which might be contributing to the phenotype of resistance observed in these animals. Previous studies have demonstrated the requirement of Th1-type cytokine profile to induce protection against *Brucella* infection [40,41]. IL-1β plays an important role in the defense against pathogens and also in the maintenance of the intestinal homeostatic balance and in the regeneration of the epithelium [59,60]. High levels of this cytokine are found in the intestinal mucosa in a normal state (steady-state), implying its importance in maintaining the mucosal barrier and in immune monitoring. The decrease in IL-1β levels observed in WT-infected mice corroborates the data of altered intestinal permeability through infection, suggesting that ST2 might have a regulatory role of this cytokine and consequently a function in the maintenance of intestinal permeability. Several studies have proposed that cell injury or death are the dominant mechanisms through which the IL-33 reaches the extracellular environment. Therefore, in a steady state, the IL-33 is not actively secreted by cells [35,61], being an important tool for the immune system, when there is a violation in the integrity of the mucosa, secondary to damage to the epithelial cells [61]. In this study, we observed that production of this cytokine in the small intestine was naturally higher in WT mice, compared to the knockout animals, suggesting that the change in intestinal permeability induced by oral infection was not through tissue damage, but via other mechanisms that need to be investigated.

The increase in MPO and EPO as an indirect measurement of neutrophils and eosinophils in the intestine of WT mice might contribute to the establishment of infection. Since *Brucella abortus* is an intracellular pathogen, and it is already described in the literature that neutrophils infected with *Brucella* are readily phagocytized by macrophages and replicate extensively within these cells, neutrophils then end up serving as “Trojan horse” vehicles for efficient bacterial dispersion, intracellular replication, and establishment of chronic infections [62]. In ST2 knockout mice, MPO and EPO were decreased after infection when compared to WT animals, which might contribute to the initial resistance profile exhibited by these mice, since there are less infected granulocytes that can carry the pathogen to spread into other host cells and organs. The absence of ST2 increased the bactericidal activity of neutrophils and macrophages against *Staphylococcus aureus* in a sepsis model [63], by increasing the production of nitric oxide of these cells. Thus, we sought to investigate whether, in an in vitro scenario, macrophages would show higher production of NO against *Brucella* infection. We observed that nitric oxide production rate is similarly influenced in WT and ST2^−/−^ macrophages, either through stimulation with *B. abortus* or LPS, in the presence or absence of IFN-γ, suggesting that bone marrow-derived macrophages have the same microbicidal potential, and that ST2 in the context of *B. abortus* infection is not involved in the regulation of NO production by these cells.

In summary, we observed that lack of ST2 is important in the model of *Brucella* oral infection but not when the animals are infected by the intraperitoneal route. In this study, we revealed that the oral infection by *Brucella abortus* alters the intestinal homeostasis in favor of its invasion and establishment of systemic infection, and the mechanisms involved in this process were partially dependent on the ST2 receptor. The ST2 receptor proved to be important in maintaining the epithelial barrier and in the negative regulation of the inflammatory immune response to oral infection through *B. abortus*.

## 4. Materials and Methods

### 4.1. Mice

Wild-type Balb/C (WT) mice were purchased from the Federal University of Minas Gerais (UFMG), and ST2 KO (kindly provided by Dr. José Carlos Alves-Filho, Department of Pharmacology, Ribeirao Preto Medical School, University of Sao Paulo, Brazil). Genetically deficient and control mice were maintained at our facilities and used at 6–8 weeks of age. Mice were housed in filter-top cages and provided with sterile water and food ad libitum. Groups of 5 to 7 animals were used to perform all experiments. The procedures for animal experimentation were approved by the Ethics Committee for the Use of Animals of the Federal University of Minas Gerais—CEUA/UFMG under protocol number 273/2017.

### 4.2. Bacteria

*Brucella abortus* smooth virulent strain 2308 was obtained from our laboratory collection. Frozen stocks were prepared from isolated colonies previously grown in *Brucella* broth medium (BB) + 1.5% agar for 3 days. One day prior to infection, *B. abortus* was grown in liquid BB and the OD was measured in a spectrophotometer. In all experiments performed in this study, OD_600_ 1 = 3 × 10^9^ CFU/mL.

### 4.3. Bacterial Counting in B. abortus Infected Mice

Five to seven mice from each group (Balb/c or ST2^−/−^) were infected orally by intragastric gavage with 1 × 10^9^ or intraperitoneally (i.p.) with 1 × 10^6^ virulent *B. abortus* S2308 in 100 µL of PBS. After 3 or 14 days post-infection, mice were sacrificed and liver and spleens were used to determine the number of bacteria through CFU counting. All organs harvested from each animal were weighed and macerated in saline (NaCl 0.9%). To determine bacterial burden, livers and spleens were serially diluted in saline and plated in duplicates on BB agar. Plates were incubated for 3 days at 37 °C and the CFU number was determined.

### 4.4. Intestinal Permeability Assay

The in vivo intestinal permeability assay to verify the barrier function was performed using the FITC-labeled Dextran method with minor modifications [64]. Briefly, food and water were removed and, after 3 h, mice were weighed and received intragastric inoculation of FITC-Dextran (0.6 mg/g body weight, PM 4000; Sigma-Aldrich, St. Louis, MO, USA). Four hours after gavage, the animals were anesthetized with ketamine/xylazine (Syntec, São Paulo, Brazil) (0.6 mL of ketamine at the concentration of 100 mg/mL, 0.4 mL of xylazine at the concentration of 20 mg/mL, and 4 mL of saline), blood was taken by cardiac puncture and was subsequently euthanized. Blood was centrifugedat 10,000 rpm for 3 min at 4 °C and serum collected was pipetted in the volume of 100 μL/well, in a plate of 96 wells (Nunc, Thermo Fisher Scientific, Norcross, GA, USA). The measurement of the fluorescence intensity of each sample (excitation, 492 nm; emission 525 nm; Synergy2, Bio Tek Instruments, Inc., Winooski, VT, USA) was performed. The measurement of intestinal permeability was expressed as the mean of the fluorescence unit. Increased fluorescence in the serum indicated increased intestinal permeability.

### 4.5. Measurement of Myeloperoxidase (MPO) and Eosinophilic Peroxidase Activity (EPO)Activity 

The evaluation of the MPO and EPO enzyme activity was used as an indirect index of neutrophil and eosinophil recruitment in the tissues, respectively. The protocol for dosage of this enzyme in homogenized tissues was performed with some modifications [65]. In brief, fragments of small intestine (100 mg) of the animals were removed and frozen at –80 °C. After thawing, the tissue was homogenized in 4.7 pH buffer (0.1 M NaCl, 0.02 M NaH_2_PO_4_.1H_2_O, 0.015 M Na_2_-EDTA) (100 mg of tissue in 1.0 mL buffer), using a tissue homogenizer, centrifuged at 10,000 rpm for 15 min at 4 °C and the precipitate was submitted to hypotonic lysis (500 μL of 0.2% NaCl solution followed by addition of equal volume of solution containing 1.6% NaCl and 5% glucose, 30 s after) for RBC lysis. After further centrifugation, the precipitate was resuspended in 0.05 M NaH_2_PO_4_ buffer (pH 5.4) containing 0.5% hexadeciltrimethylammonium bromide (HTAB) (Sigma) and was re-homogenized. Aliquots of 1 mL of the suspension were transferred to microcentrifuge tubes of 1.5 mL and submitted to three freezing/thawing cycles using liquid nitrogen. These samples were again centrifuged for 15 min at 10,000 rpm. The supernatant was collected and MPO activity was calculated by measuring the changes in optical density (OD) at 450 nm, using tetramethylbenzidine (TMB) (1.6 mM) (Sigma) and H_2_O_2_ (0.5 mM). The supernatant was also used to quantify the peroxidase activity. The assay was performed in 96-well plates, 75 μL per sample or blank well (PBS/HTAB 0.5%) was incubated with 75 μL of substrate (o-phenylenediamine (OPD) (Sigma) 1.5 mM, in Tris-HCl buffer—0.075 μM, pH 8, supplemented with H_2_O_2_ 6.6 mM). The plate was incubated at 20 °C in the dark for approximately 30 min and the reaction was interrupted by the addition of 50 μL of H_2_SO_4_ 1M. The reaction was measuredin a microplate reader (Multiskan FC Thermo Scientific, Norcross, GA, USA) with a 492 nm filter.

### 4.6. Measurement of Cytokine Concentrations

To evaluate the production of cytokines, fragments of the small intestine with approximately 100 mg were homogenized using a tissue homogenizer (T10 Basic ULTRA-TURRAX^®^, IKA, Königswinter, Germany) in 1 mL of cytokine extraction solution—PBS containing antiprotease cocktail (0.1 mM PMSF, 0.1 mM benzethonium chloride, 10 mM EDTA, and 20 KI aprotinin A) and 0.05% Tween-20. Then, the homogenates were centrifuged at 4 °C for 10 min at 10,000 rpm. The supernatants were immediately collected and stored at –80 °C for subsequent measurement. The concentrations of IL-1β, IL-33, IFN-γ, TNF-α, and IL-10 was performed through the ELISA method, using kits purchased from R&D Systems (DuoSet) (R&D Systems, Minneapolis, MN, USA) according to manufacturers’ recommendations.

### 4.7. Real-Time PCR (RT–PCR)

RNA was extracted from small intestine with TRIzol reagent (Invitrogen, Thermo Fisher Scientific, Norcross, GA, USA) according to the manufacturer’s instructions. cDNA was synthesized by reverse transcription (RT) from 1 µg of total RNA and was used to perform RT–PCR in a final volume of 10 µL containing SYBR green PCR Master Mix (Applied Biosystems, Carlsbad, CA, USA) and 20 µM of primers. RT–PCR was performed in triplicates, on an ABI 7900 Real-time PCR system (Applied Biosystems). The primers used for gene amplification were as follows: 18S forward 5′-CGTTCCACCAACTAAGAACG-3′, reverse 5′-CTCAACACGGGAAACCTCAC-3′; MUC2 forward 5′- CACCAACACGTCAAAAATCG -3′,reverse 5′- CGCAGAACTCCCAGTAGCA -3′; Amphiregulin forward 5′- GCCATTATGCAGCTGCTTTGGAGC -3′, reverse 5′- TGTTTTTCTTGGGCTTAATCACCT -3′; ZO-1 forward 5′-TGAACGCTCTCATAAGCTTCGTAA-3′, reverse 5′-ACCGTACCAACCATCATTCATTG-3′; ZO-2 forward 5′-CCATGGGCGCGGACTATCTGA-3′, reverse 5′-CTGTGGCGGGGAGGTTTGACTTG-3′, ZO-3 forward 5′-AAGCACGCAATCCTGGATGTCACC-3′, reverse 5′-GTCGCGCCTGCTGTTGCTGTATTA-3′; claudin-1 forward 5′-AGCCAGGAGCCTCCCCCGCAGCTGCA-3′, reverse 5′-CGGGTTGCCTGCAAAGT-3′. The levels of mRNAs are presented as relative expression units after normalization to 18S transcripts.

### 4.8. Generation of BMDMs

Bone-marrow cells were obtained from femur and tibiae of ST2 KO and WT mice and they were differentiated into BMDMs using a previously described protocol, with some modifications [66]. In brief, cells were seeded on 24-well plates at 5 × 10^5^ cell/mL (day 0) and maintained in DMEM medium containing 10% FBS, 100 U/mL penicillin, 100 μg/mL streptomycin, and 20% LCCM (L929-conditioned medium), at 37 °C in a 5% CO_2_ atmosphere for 7 days. On day 4 of incubation, the medium was fully replaced. Four hours before stimulation or infection, BMDMs were maintained only in the DMEM medium containing 1% FBS.

### 4.9. Nitrite Measurement by Griess Reagent 

The nitric oxide assay was performed as described previously [15]. The concentration of nitrite (NO_2_^–^), a stable metabolite of NO, was measured using Griess reagent (1% sulfanilamide and 0.1% naphthylethylenediaminedihydrochloride in 2.5% phosphoric acid). In brief, 50 μL of cell culture supernatants was mixed with 50 µL of Griess reagent. Subsequently, the mixture was incubated, protected from light at room temperature for 5 min, and the absorbance at 550 nm was measured in a microplate reader. Fresh culture medium (DMEM + 1% FBS) was used as a blank in every experiment. The quantity of nitrite was determined from a sodium nitrite (NaNO_2_) standard curve.

### 4.10. Gut Pathology

The small intestine of the animals was removed soon after the sacrifice, and the duodenum was separated for histological analysis. The tissues were extended in contact with the filter paper and opened by removing all their contents without damaging the mucosa. The fragments were transferred to a container containing 10% formaldehyde solution for a short period for pre-fixing. The prefixed material was placed on a flat surface and wound in a spiral with the mucosa facing inwards to form rolls. The rolls were tied with line and fixed by immersion in 10% formalin solution in PBS, pH 7.4 for 48 h, and embedded in paraffin. One 4-μm-thick sections were obtained and stained with hematoxylin-and-eosin (H&E) and examined under light microscopy by two pathologists blinded to the experiment. Measurement of villus heights, crypt, and total mucosa thickness depth was performed using the ImageJ software. Fifteen intact and well-oriented villi, crypts, and total mucosa thickness were measured from each animal of each mouse group (*n* = 5).

### 4.11. Statistical Analysis

The experiments were repeated at least twice with similar results. Graphs and data analysis were performed using GraphPad Prism 5 (GraphPad Software, San Diego, CA, USA), using one-way ANOVA followed by a post-test of Student-Newman-Keuls.

## Supplementary Materials

The following are available online at https://www.mdpi.com/2076-0817/9/5/328/s1, Figure S1: ST2 receptor deficiency does not influence the production of IFN-γ and TNF-α in liver after *Brucella abortus* infection.
