# Supplementary material for: The Role of ST2 Receptor in the Regulation of Brucella abortus Oral Infection
Source: Pathogens. 2020 Apr 28;9(5):328. doi: 10.3390/pathogens9050328 (PMC7281115; doi:10.3390/pathogens9050328)
Supplement: Supplementary file 1 [file pathogens-09-00328-s001.pdf]

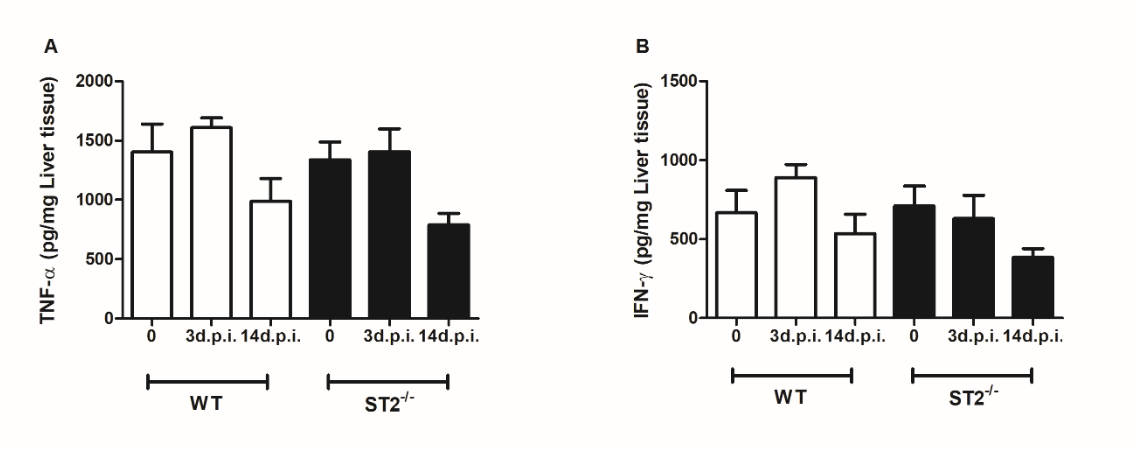

Supplementary Figure 1. ST2 receptor deficiency does not influence the production of IFN-γ and TNF-α in liver after *Brucella abortus* infection. WT and $ST2^{-/-}$ mice were infected orally with $1x10^9$ CFU of *B. abortus* and after 3 or 14 days of infection liver samples were assessed for cytokine production, such as TNF-α (A), IFN-γ (B) by ELISA. Results expressed as mean ± standard deviation (n = 5).
